# A New Clinical Model to Estimate the Pre-Test Probability of Cushing’s Syndrome: The Cushing Score

**DOI:** 10.3389/fendo.2021.747549

**Published:** 2021-10-05

**Authors:** Mirko Parasiliti-Caprino, Fabio Bioletto, Tommaso Frigerio, Valentina D’Angelo, Filippo Ceccato, Francesco Ferraù, Rosario Ferrigno, Marianna Minnetti, Carla Scaroni, Salvatore Cannavò, Rosario Pivonello, Andrea Isidori, Fabio Broglio, Roberta Giordano, Maurizio Spinello, Silvia Grottoli, Emanuela Arvat

**Affiliations:** ^1^ Endocrinology, Diabetes and Metabolism, Department of Medical Sciences, University of Turin, Turin, Italy; ^2^ Oncological Endocrinology, Department of Medical Sciences, University of Turin, Turin, Italy; ^3^ Endocrinology Unit, Department of Medicine, DIMED, Hospital-University of Padova, Padova, Italy; ^4^ Dipartimento di Patologia Umana DETEV “G. Barresi”, Università di Messina, UOC di Endocrinologia, AOU Policlinico G. Martino, Messina, Italy; ^5^ Sezione di Endocrinologia, Dipartimento di Medicina Clinica e Chirurgia, Università Federico II di Napoli, Naples, Italy; ^6^ Department of Experimental Medicine, Sapienza University of Rome, Rome, Italy; ^7^ Department of Biological and Clinical Sciences, University of Turin, Turin, Italy; ^8^ Novartis Farma, Origgio, Italy

**Keywords:** cortisol, adrenocorticotrophic hormone, hypercortisolism, pituitary disease, adrenal disease, corticosteroids

## Abstract

**Background:**

Hypercortisolism accounts for relevant morbidity and mortality and is often a diagnostic challenge for clinicians. A prompt diagnosis is necessary to treat Cushing’s syndrome as early as possible.

**Objective:**

The aim of this study was to develop and validate a clinical model for the estimation of pre-test probability of hypercortisolism in an at-risk population.

**Design:**

We conducted a retrospective multicenter case-control study, involving five Italian referral centers for Endocrinology (Turin, Messina, Naples, Padua and Rome). One hundred and fifty patients affected by Cushing’s syndrome and 300 patients in which hypercortisolism was excluded were enrolled. All patients were evaluated, according to current guidelines, for the suspicion of hypercortisolism.

**Results:**

The Cushing score was built by multivariable logistic regression, considering all main features associated with a clinical suspicion of hypercortisolism as possible predictors. A stepwise backward selection algorithm was used (final model AUC=0.873), then an internal validation was performed through ten-fold cross-validation. Final estimation of the model performance showed an average AUC=0.841, thus reassuring about a small overfitting effect. The retrieved score was structured on a 17.5-point scale: low-risk class (score value: ≤5.5, probability of disease=0.8%); intermediate-low-risk class (score value: 6-8.5, probability of disease=2.7%); intermediate-high-risk class (score value: 9-11.5, probability of disease=18.5%) and finally, high-risk class (score value: ≥12, probability of disease=72.5%).

**Conclusions:**

We developed and internally validated a simple tool to determine pre-test probability of hypercortisolism, the Cushing score, that showed a remarkable predictive power for the discrimination between subjects with and without a final diagnosis of Cushing’s syndrome.

## Introduction

Endogenous Cushing’s syndrome (CS) is defined as a complex of signs and symptoms resulting from chronic and excessive exposure to glucocorticoids (1). It is usually characterized by the loss of the normal feedback mechanism of the hypothalamic-pituitary-adrenal (HPA) axis and the circadian rhythm of cortisol (2, 3). Due to the variable pattern of the biochemical parameters and the low specificity of clinical manifestations (4), the diagnosis of CS is often a challenge for clinicians (5, 6). However, because of its associated burden of morbidity (7–9) and mortality (10–13), it is necessary to diagnose endogenous hypercortisolism as early as possible (12). A recent study showed that the time from the beginning of symptoms to the actual diagnosis may extend up to 4 years, and that on average 4.6 physicians may need to be consulted before a correct recognition of the disease (14). The Endocrine Society (ES) guidelines for CS recommend the initial use of at least one of the following tests: 24-hour urinary free cortisol (UFC), late-night salivary cortisol (LNS-F), 1 mg overnight or 2 mg 48-hour dexamethasone suppression test (DST) (15). Although all these tests proved to be quite accurate (16, 17), discordant results are still possible and not uncommon, even after the introduction of mass-spectrometry-based methods (18, 19). Therefore, many authors emphasize the key importance of estimating clinical probability of CS prior to the initiation of biochemical work-up (20).

Cipoli et al. (21) encouraged the application of a Bayesian approach for the diagnosis of CS, in which any additional test result modifies the probability of hypercortisolism, rather than providing a dichotomous diagnostic answer. In particular, these authors proposed the use of a Fagan nomogram, in which post-test probability is calculated on the basis of pre-test clinical probability and of test-specific likelihood ratio. This Bayesian approach to the CS diagnostic work-up is methodologically well-grounded and allows a more efficient handling and interpretation of biochemical test results, but it poses a great problem, i.e. a reliable quantitative estimate of clinical pre-test CS probability based on initial presenting features.

Differently from acromegaly (22), nowadays there is no shared, accurate and standardized model to estimate pre-test probability of CS (20). Most authors, as well as the ES guidelines (15), do not provide a defined and reproducible method to retrieve a quantitative estimate of clinical pre-test CS probability, leaving the distinction between “low-risk” and “high-risk” to the clinical judgement.

Only few attempts to provide a score for the assessment of clinical CS probability have been proposed so far. Nugent et al. (23) proposed a clinical score to discriminate patients with hypercortisolism, but its diagnostic performance was quite low. More recently, Leon-Justel et al. (24) developed another scoring system for CS risk prediction. However, the model proposed by the authors comprised both clinical parameters and LNS-F, thus it was not strictly applicable to the purpose of a purely clinical estimate of pre-test CS probability. The model performance achieved by included only clinical parameters was also evaluated, but was again quite low. Finally, the statistical methods adopted for score development and validation have been thoroughly criticized, further limiting the reliability of its results (25).

Thus, the aim of this study was to develop and validate a standardized and accurate clinical model (the Cushing score) for the estimation of pre-test probability of CS in an at-risk population.

## Materials and Methods

### Patient Selection

Data of patients who underwent biochemical testing for clinical suspicion of CS (patients with multiple and progressive features predictive of CS; with unusual features for age, such as osteoporosis and hypertension and/or with adrenal incidentaloma compatible with adenoma) in five Italian tertiary referral centers (Turin, Messina, Naples, Padua, Rome) were collected from prospective registries and analyzed retrospectively. In order to reduce the possible heterogeneity between centers and physicians in the criteria for the initiation of a CS biochemical work-up, only patients presenting at least two features of metabolic syndrome according to NCEP ATP-III criteria (26) were included.

Exclusion criteria were the lack of sufficient data for baseline clinical feature assessment and/or the non-completion of the diagnostic work-up for the final confirmation or exclusion of CS.

According to a case-control study design, a pre-established number of 150 consecutive patients in which CS was confirmed were enrolled as the case group; a pre-established number of 300 consecutive patients in which CS was excluded were enrolled as the control group.

Approval from local ethics committees was obtained for the analysis of patient data in all centres with a central coordination by the Ethics Commitee of the City of Health and Science University Hospital of Turin. Written informed consent from patients was obtained in all centres.

### Data Collection

For each patient, all main baseline characteristics potentially associated with CS were collected, including personal data and hypercortisolism-related clinical signs, symptoms or comorbidities, such as skin changes (facial plethora, purple striae, easy bruisability, hirsutism and/or seborrhoea), muscle wasting (proximal muscle atrophy, proximal muscle weakness), atypical fat distribution (facial fullness, dorsocervical fat pad, central adiposity), cardiometabolic alterations (obesity, diabetes, dyslipidemia, hypertension), bone mineral loss (osteopenia or osteoporosis) and psychiatric symptoms. In order to limit the possibility of recall bias, only data reported in clinical registries prior to the beginning of hormonal work-up were examined for baseline feature extraction.

In addition, for each patient, the final and definite conclusion of the diagnostic work-up (confirmation or exclusion of CS) was also recorded. The diagnosis was performed in all cases by a full biochemical assessment and clinical follow-up, according to the recommendations of current international guidelines (15).

### Statistical Analysis

The study followed the TRIPOD statement for Transparent Reporting of a multivariable prediction model for Individual Prognosis Or Diagnosis (27, 28).

Baseline characteristics of all patients included in the analysis were summarized using mean and standard deviation for continuous variables and percent values for binary and categorical data. Between-group differences were evaluated by Student t-test for continuous variables and chi-squared test for categorical variables.

All clinical variables reported in data extraction were included in a multivariable logistic regression model. A stepwise backward selection algorithm was used in order to retain in the model only the variables showing an independent meaningful correlation, defined by a p-value <0.10, with the outcome of interest. The final model performance was evaluated by the area under curve (AUC) at ROC analysis.

A ten-fold cross-validation algorithm was adopted for internal validation, in order to provide an estimate of model performance on unseen data. After a random split of the original sample into ten groups, the modelling process was entirely repeated from variable selection in nine of them, and its performance was evaluated in the tenth. The process was then repeated ten times, rotating the validation group at each round. Final model performance was obtained as the average performance over the ten iterations.

In order to simplify the use of the model in clinical practice, a weighted risk score was created upon normalization and rounding of regression β-coefficients to the nearest integer or half-integer value.

Statistical analysis was performed using STATA 16 (StataCorp, College Station, Texas, USA).

## Results

### General Characteristics of the Study Population

One hundred and fifty patients with confirmed CS were enrolled as the case group and 300 patients in which CS was excluded were enrolled as the control group (**Table 1** summarizes the differences in baseline characteristics between the two groups). One hundred and fourteen patients (76.0%) were affected by Cushing’s disease, 37 (19.3%) by ACTH-independent CS (35 adrenocortical adenomas and 2 cancers) and 7 (4.6%) by ectopic hypercortisolism.

**Table 1:** Baseline clinical characteristics in patients diagnosed with Cushing’s syndrome (cases) and patients in which Cushing’s syndrome was excluded (controls).

Variables/parameters	Cases (N = 150)	Controls (N = 300)	p-value
Age	42.6 ± 1.1	44.9 ± 1.0	0.129
Age category			0.018
≥60 years	15 (10.0%)	62 (20.7%)	
40-59 years	71 (47.3%)	125 (41.6%)	
<40 years	64 (42.7%)	113 (37.7%)	
Female sex	123 (82.0%)	216 (72.0%)	0.020
Facial fullness	101 (67.3%)	83 (27.7%)	<0.001
Facial plethora	68 (45.3%)	46 (15.3%)	<0.001
Purple striae	49 (32.7%)	48 (16.0%)	<0.001
Easy bruisability	47 (31.3%)	30 (10.0%)	<0.001
Proximal muscle atrophy	74 (49.3%)	34 (11.3%)	<0.001
Proximal muscle weakness	53 (35.3%)	50 (16.7%)	<0.001
Hirsutism and/or seborrhoea	69 (46.0%)	98 (32.7%)	0.006
Psychiatric symptoms	62 (41.3%)	69 (23.0%)	<0.001
Dorsocervical fat pad	81 (54.0%)	60 (20.0%)	<0.001
Central adiposity	206 (68.9%)	108 (72.0%)	0.468
Obesity (BMI ≥ 30 kg/m^2^)	54 (36.0%)	192 (64.0%)	<0.001
Hypertension	106 (70.7%)	153 (51.0%)	<0.001
Diabetes	54 (36.0%)	73 (24.3%)	0.010
Dyslipidemia	91 (60.7%)	147 (49.0%)	0.019
Bone mineral density			<0.001
Normal	70 (46.7%)	242 (80.7%)	
Osteopenia	32 (21.3%)	33 (11.0%)	
Osteoporosis	48 (32.0%)	25 (8.3%)	

Patients with CS were characterized by a non-significant trend towards younger age when considered as a continuous measure (42.6 ± 1.1 *vs* 44.9 ± 1.0 years, p=0.129), which became statistically significant upon categorization (p=0.018). The case group also showed a higher prevalence of female gender (82.0% *vs* 72.0%, p=0.020).

Almost all hypercortisolism-related clinical signs, symptoms or comorbidities showed a significantly higher prevalence in the case group compared to the control one (**Table 1**). The only exceptions were central adiposity, for which no significant difference could be noted (68.9% *vs* 72.0%, p=0.468), and obesity, for which an inverse association was found (36.0% *vs* 64.0%, p<0.001).

### Model Construction and Internal Validation

A clinical prediction model for the diagnosis of CS was built by multivariable logistic regression. All variables reported in **Table 1** were initially considered as possible predictors and included in the model. Then, a stepwise backward selection algorithm was used in order to retain in the model only the variables showing an independent meaningful correlation, defined by a p-value < 0.10, with the outcome of interest.

The final multivariable logistic regression model retrieved through this approach is reported in **Table 2**. The predictors that were retained after stepwise backward selection were age (OR 3.15, 95% CI 1.34-7.42, p=0.009 for age 40-59 years; OR 7.35, 95% CI 2.79-19.37, p< 0.001 for age < 40 years), facial fullness (OR 2.13, 95% CI 1.16-3.93, p=0.015), facial plethora (OR 1.98, 95% CI 1.04-3.77, p=0.038), proximal muscle atrophy (OR 2.46, 95% CI 1.24-4.88, p=0.010), hirsutism and/or seborrhoea (OR 1.91, 95% CI 1.06-3.41, p=0.030), absence of obesity (OR 5.93, 95% CI 3.27-10.73, p<0.001), hypertension (OR 3.36, 95% CI 1.81-6.21, p<0.001), diabetes (OR 1.87, 95% CI 0.98-3.57, p=0.059), and bone mineral density (OR 2.35, 95% CI 1.14-4.86, p=0.021 for osteopenia; OR 5.13, 95% CI 2.39-11.02, p<0.001 for osteoporosis).

**Table 2:** Cushing’s syndrome prediction by multivariable logistic regression model.

Predictor	OR	95% CI	p-value
Age			
≥ 60 years	1.00		
40-59 years	3.15	1.34-7.42	0.009
< 40 years	7.35	2.79-19.37	<0.001
Facial fullness	2.13	1.16-3.93	0.015
Facial plethora	1.98	1.04-3.77	0.038
Proximal muscle atrophy	2.46	1.24-4.88	0.010
Hirsutism and/or seborrhoea	1.91	1.06-3.41	0.030
Dorsocervical fat pad	2.27	1.19-4.32	0.013
Non-obesity (BMI < 30 kg/m^2^)	5.93	3.27-10.73	<0.001
Hypertension	3.36	1.81-6.21	<0.001
Diabetes	1.87	0.98-3.57	0.059
Bone mineral density			
Normal	1.00		
Osteopenia	2.35	1.14-4.86	0.021
Osteoporosis	5.13	2.39-11.02	<0.001

The predictive performance of the overall model was assessed by the calculation of the AUC at ROC analysis, which was equal to 0.873.

Internal validation of the model was performed through ten-fold cross-validation, as already described in the section about statistical analysis. As recommended by the TRIPOD statement, the whole process of model construction was entirely repeated, starting from variable selection, at each round. The final estimation of the model performance on unseen data, obtained as the average AUC over the ten iterations, was equal to 0.841, thus reassuring about a small overfitting effect.

### Score Retrieval and Risk Class Stratification

**Figure 1:**
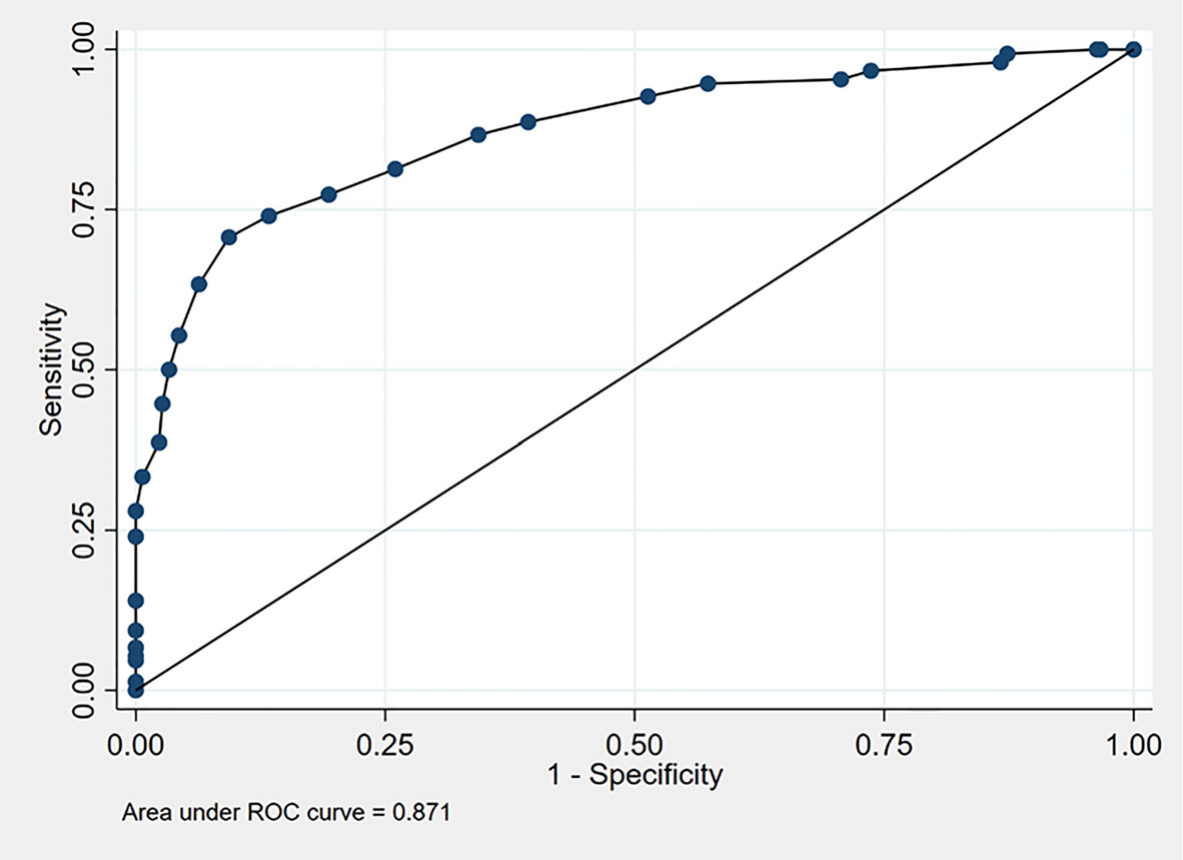
ROC curve of the Cushing score.

In order to simplify the use of the model in clinical practice, integer or half-integer point scores were assigned to each predictor upon normalization and rounding of regression β-coefficients, as reported in **Table 3**. Notably, this mild simplification did not lead to a significant reduction in the predictive power of the model, since the AUC only slightly declined from 0.873 to 0.871 (**Figure 1**).

**Table 3:** Cushing score point assignment according to multivariable regression coefficients.

Parameter	β-coefficient	Points for Cushing score
Age		
*40-59 years*	+1.147	2
*<40 years*	+1.995	3
Facial fullness	+0.758	1
Facial plethora	+0.684	1
Proximal muscle atrophy	+0.900	1.5
Hirsutism and/or seborrhoea	+0.646	1
Dorsocervical fat pad	+0.819	1.5
Non-obesity (BMI < 30 kg/m^2^)	+1.779	3
Hypertension	+1.211	2
Diabetes	+0.625	1
Bone mineral density		
*Osteopenia*	+0.856	1.5
*Osteoporosis*	+1.636	2.5

According to the assigned coefficients, the retrieved score was structured on a 17.5-point scale. **Table 4** illustrates the stratification of patients according to score risk classes and eventual CS diagnosis. Patients were grouped in classes of 2-point width, with the exception of the central ones which were further split for finer stratification. The likelihood ratio (LR) for CS diagnosis was computed for each class. The correspondent clinical estimate of CS probability was, thus, derived assuming a case prevalence of 5%, which is consistent with previous reports in literature for at-risk populations ^10,15,16,17^.

**Table 4:** Cushing score distribution in the case group and in the control group, according to pre-specified risk classes; likelihood-ratios for CS diagnosis for each risk class; estimate of CS clinical probability for each risk class, assuming a case prevalence of 5%.

Cushing score	Cases (N = 150)	Controls (N = 300)	Likelihood ratio	Estimate of CS clinical probability*	Pooled likelihood ratio	Pooled estimate of CS clinical probability*
0.0-1.5 pts	0	0	–	–	0.15	0.8%
2.0-3.5 pts	3	40	0.15	0.8%		
4.0-5.5 pts	8	106	0.15	0.8%		
6.0-6.5 pts	9	51	0.35	1.8%	0.52	2.7%
7.0-7.5 pts	14	45	0.62	3.2%		
8.0-8.5 pts	10	30	0.67	3.4%		
9.0-9.5 pts	23	15	3.07	13.9%	4.31	18.5%
10.0-10.5 pts	16	5	6.40	25.2%		
11.0-11.5 pts	17	6	5.67	23.0%		
12.0-13.5 pts	36	2	36.00	65.5%	50.00	72.5%
14.0-15.5 pts	12	0	+∞	100.0%		
16.0-17.5 pts	2	0	+∞	100.0%		

*Assuming a case prevalence of 5%.

For a simpler clinical application, a broader clustering of risk classes was proposed, according to whether the correspondent LR was less than 0.2 (low risk), comprised between 0.2 and 1 (intermediate-low risk), comprised between 1 and 10 (intermediate-high risk), or greater than 10 (high-risk). The low-risk class (Cushing score: 0-5.5) corresponded to a clinical estimate of CS probability of 0.8%. The intermediate-low-risk class (Cushing score: 6.0-8.5) accounted for a CS probability of 2.7%. The intermediate-high-risk class (Cushing score: 9.0-11.5) had a CS probability of 18.5%. Finally, patients in the high-risk class (Cushing score: 12.0-17.5) showed a CS probability of 72.5%.

## Discussion

In the present multicenter study, we developed and internally validated a multivariable prediction model for the estimation of the clinical probability of CS in at-risk populations. To facilitate its clinical use, we also derived a simplified scoring system, the Cushing score, by assigning integer or half-integer scores to each of the included predictors. Our model, based solely on clinical data, showed a remarkable predictive power for the discrimination between subjects with and without a final diagnosis of CS, with an AUC of 0.873. Considering the high clinical impact of CS, we proposed a Cushing score cut-off >6 for submitting patients to first-line tests for hypercortisolism, particularly in settings of high suspicion or in patients referred to third level centers. However, in some cases of low suspicion, it could be considered the adoption of a Cushing score cut-off ≥9.

Even if a recent meta-analysis (17) suggested that all the three first-line tests have a reliable accuracy in the diagnosis of CS, the determination of the pre-test probability of hypercortisolism is crucial. Moreover, the pitfalls of each of these tests should be considered, particularly when they are performed, often inappropriately or using no accurate methods of evaluation, in a low-risk population. This approach is different from what is usually considered in the literature as the screening for CS, which is important to remember that is unjustified for this endocrine disease (29) and is defined as tests done among the asymptomatic people to identify those at an increased risk of a disease or disorder. Those identified are sometimes then offered a subsequent diagnostic test or procedure, or, in some instances, a treatment or preventive medication (30). On the contrary, looking for additional illnesses in those with medical problems is termed case finding, which is a completely different medical approach. In the case detection of CS, the abnormality of first-line tests suggests the diagnosis of endogenous hypercortisolism. Therefore, the application of a clinical score able to accurately estimate the baseline probability of CS would be therefore helpful in a twofold way: (a) for a better selection of at-risk patients that should be submitted to biochemical investigations, thus avoiding unnecessary testing and reducing the burden of inaccurate diagnostic test results; (b) for a more conscious interpretation of first-line biochemical test results in patients who were submitted to, thus favouring a better subsequent work-up especially in case of contrasting first-line test results. Even if other ways to early identify patients with acromegaly (31, 32) and CS (33, 34) have been proposed, such as the face classification, the advantage of a score is indeed in its cheap and simple application.

The accuracy of the Cushing score obtained in our study is considerably better than the one obtained by Leon-Justel et al. (24), as their model – when considering only clinical data – showed an AUC of 0.684. Moreover, the performance of our clinical model is not far from the one obtained by the same authors by combining clinical data with LNS-F (AUC equal to 0.916). This remarkable improvement in terms of predictive power with respect to the clinical model proposed by Leon-Justel et al. (24) likely derives from the inclusion, in our sample cohort, of a significantly greater number of patients with CS (150 patients vs 26 patients). This enhanced the possibility to obtain statistically significant results for a higher number of predictors and, therefore, finally allowed the inclusion in the final model of a higher number of parameters.

At univariate analysis, almost all hypercortisolism-related clinical signs, symptoms or comorbidities showed a significantly higher prevalence in the case group compared to the control group, with the only exceptions of central adiposity and obesity. These latter results might seem to be paradoxical at first sight, but were not actually fully surprising. In fact, patients who are usually addressed to a biochemical work-up for CS suspicion are often patients with the clinical features of the metabolic syndrome, of which central adiposity and obesity are probably the main hallmarks; this was probably further emphasized in our study, due to the adopted inclusion criteria. Notably, these results were also in line with the findings by Leon-Justel et al. (24), which showed – similarly to our study – a significantly higher prevalence of obesity in patients without CS than in those with it.

The absence of obesity was retained as a significant independent predictor of CS also in our final multivariable model. This result might seem contradictory, as obesity is, actually, one of the typical features of CS patients with respect to healthy controls. However, our model was developed (and it is meant to be applied) to guide the diagnostic work-up in the clinical context of at-risk populations, in which patients with metabolic syndrome are extensively represented. In this context, therefore, it is not surprising that the absence (rather than the presence) of obesity increases the likelihood of CS diagnosis, as other typical CS features such as facial fullness, dorsocervical fat pad, hypertension and diabetes are far more suspicious when diagnosed in non-obese rather than in obese subjects. It has also to be noted that the combined presence of at least 2 hypercortisolism-related complications (such as hypertension, diabetes and osteoporosis/osteopenia) at young age (< 40 years) results in a score value, which indicates to test patients for CS, according to the international guidelines (15).

The strengths of our study are the longstanding expertise of the involved centers in diagnosis and treatment of CS, the high number of cases and controls involved in the study and the internal validation of the model. Moreover, as discussed and recognized in a recent expert review (20), our control group is the ideal one for a clinical score development, since it is represented by patients with an initial clinical suspicion for hypercortisolism in which CS was excluded. It has to be noted that in the follow-up no patients of the control group showed the appearance of further signs or symptoms of hypercortisolism.

Our study has some limitations that are worth to be discussed. The first one is related to its retrospective design; however, the retrieved data were prospectively collected and, most notably, the recall of baseline clinical features for each patient was based only on data retrieved from clinical reports preceding the beginning of any biochemical work-up for hypercortisolism; therefore, the influence of a recall bias is overall likely to be limited. A second limitation might be related to a potential selection bias, which might arise from the tertiary nature of our centers; however, the adopted criteria for patient selection ensured an overall adequate homogeneity to the series. A third limitation may be represented by the possible optimistic estimation of the model performance which may arise by the data-driven backward stepwise variable selection; however, as recommended in TRIPOD statement (28), the extent of overfitting due to the use of this variable selection strategy was estimated and accounted for in the internal validation procedure of the model.

Future perspectives include the prospective validation of the predictive capability of our score in a prospective patient cohort. In fact, even if internal validation of the model already reassured about model consistency, an external validation with prospective design would further enforce the validity of the model.

## Conclusions

This is the first study that describes an algorithm exclusively based on clinical variables and able to guide the clinician in the distinction of cases with low or high pre-test probability of CS. The derived Cushing score is a simple tool that could be extensively adopted in clinical practice and might be of significant help in reducing the length and the potential pitfalls in CS diagnostic work-up, with reflections in the improvement of patient health and in the reduction of health care costs, particularly during the COVID-19 pandemic.

## Data Availability Statement

The raw data supporting the conclusions of this article will be made available by the authors, without undue reservation.

## Ethics Statement

Approval from local ethics committees was obtained for the analysis of patient data in all centres with a central coordination by the Ethics Commitee of the City of Health and Science University Hospital of Turin. Written informed consent from patients was obtained in all centres.

## Author Contributions 

Conceptualization: CS, SC, RP, AI, FB, RG, SG, and EA. Methodology: MP-C and FB. Validation: TF and VD’A. Resources: FC, FF, RF, MM, and MS. Data Curation: MP-C, FB, and TF. Writing – Original Draft Preparation: MP-C and FB. Writing – Review & Editing: CS, SC, RP, AI, FB, RG, SG, and EA. Supervision: RG, SG, and EA. All authors contributed to the article and approved the submitted version.

## Conflict of Interest

MS was employed by Novartis.

The remaining authors declare that the research was conducted in the absence of any commercial or financial relationships that could be construed as a potential conflict of interest.

## Publisher’s Note

All claims expressed in this article are solely those of the authors and do not necessarily represent those of their affiliated organizations, or those of the publisher, the editors and the reviewers. Any product that may be evaluated in this article, or claim that may be made by its manufacturer, is not guaranteed or endorsed by the publisher.
